# Supplementary material for: Modulation of Acute Intestinal Inflammation by Dandelion Polysaccharides: An In-Depth Analysis of Antioxidative, Anti-Inflammatory Effects and Gut Microbiota Regulation
Source: Int J Mol Sci. 2024 Jan 24;25(3):1429. doi: 10.3390/ijms25031429 (PMC10855136; doi:10.3390/ijms25031429)
Supplement: Supplementary file 1 [file ijms-25-01429-s001.zip › ijms-2833956-supplementary.pdf]

Supplementary Table S1. Primer sequence list for qRT-PCR.

Gene	Forward primer (5'-3')	Reverse primer (5'-3')
Claudin-1	ACCCTGCCCGTGACTTTG	AGGAGTCGAAGACTTTGCACT
ZO-1	GATGAGCGGGCTACCTTA	TGGAGACTGCGTGGAATG
TNF-α	GGGTGTTCATCCATTCTC	GGAAAGCCCATTTGAGT
IL-22	ATGAGTTTTTCCCTTATGGGGAC	GCTGGAAGTTGGACACCTCAA
IL-6	GAAACCGCTATGAAGTTCCTCTCTG	TGTTGGGAGTGGTATCCTCTGTGA
IL-1β	GGCTGGACTGTTTCTAATGC	ATGGTTTCTTGTGACCCTGA
CAT	GAGGCGGGAACCCAATAG	GTGTGCCATCTCGTCAGTGAA
Nrf2	CTTTAGTCAGCGACAGAAGGAC	AGGCATCTTGTTTGGGAATGTG
HO-1	GAACCCAGTCTATGCCCCAC	GGCGTGCAAGGATGATTTC
NQO1	AGGATGGGAGGTACTCGAATC	AGGATGGGAGGTACTCGAATC

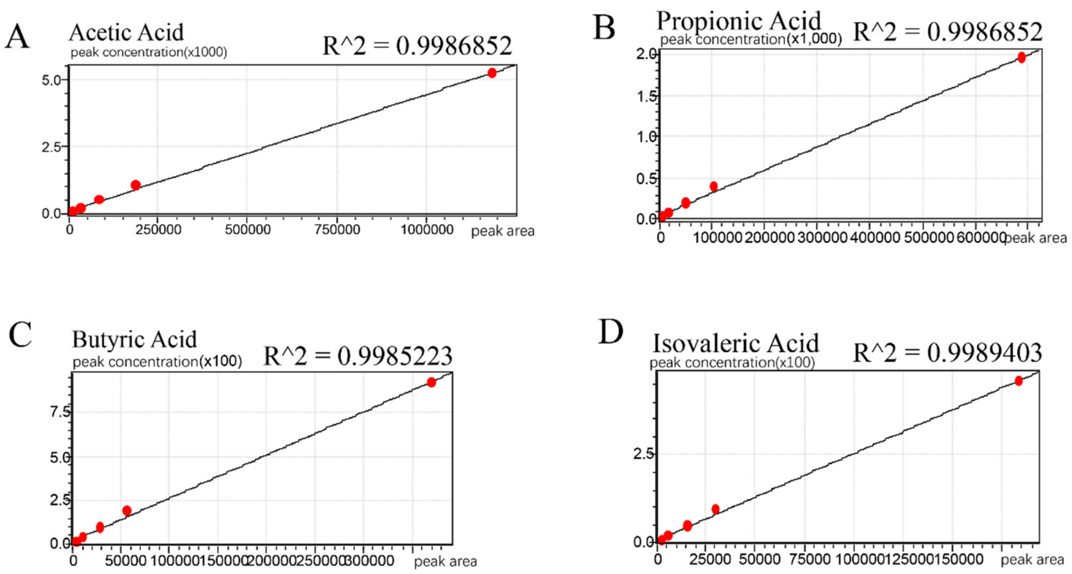

Supplement Figure S1. Peaks of chromatogram reported to standards of Acetic Acid (A), Propionic Acid (B), Butyric Acid (C) and Isovaleric Acid (D). R^2 in turn 0.9986852, 0.9986852, 0.9985223 and 0.9989403.
